# From Senescent Cells to Systemic Inflammation: The Role of Inflammaging in Age-Related Diseases and Kidney Dysfunction

**DOI:** 10.3390/cells14221831

**Published:** 2025-11-20

**Authors:** Federica De Luca, Valentina Camporeale, Giorgia Leccese, Roberto Cuttano, Dario Troise, Barbara Infante, Giovanni Stallone, Giuseppe Stefano Netti, Elena Ranieri

**Affiliations:** 1 Unit of Clinical Pathology, Department of Medical and Surgical Sciences, University of Foggia—University Hospital “Policlinico Riuniti”, Viale Luigi Pinto, 71122 Foggia, Italy; federica.deluca@unifg.it (F.D.L.); valentina.camporeale@unifg.it (V.C.); giorgia_leccese.555553@unifg.it (G.L.); roberto.cuttano@unifg.it (R.C.); elena.ranieri@unifg.it (E.R.); 2 Center for Research and Innovation in Medicine (CREATE), Department of Medical and Surgical Sciences, University of Foggia—University Hospital “Policlinico Riuniti”, Viale Luigi Pinto, 71122 Foggia, Italy; dario.troise@unifg.it (D.T.); barbara.infante@unifg.it (B.I.); giovanni.stallone@unifg.it (G.S.); 3 Unit of Nephrology, Dialysis and Transplantation, Advanced Research Center on Kidney Aging (A.R.K.A.), Department of Medical and Surgical Sciences, University of Foggia—University Hospital “Policlinico Riuniti”, Viale Luigi Pinto, 71122 Foggia, Italy

**Keywords:** inflammaging, immunosenescence, cellular senescence, chronic kidney disease (CKD), senolytics

## Abstract

**Highlights:**

**What are the main findings?**

- Inflammaging and immunosenescence are tightly interconnected processes that drive the onset and progression of major age-related diseases.
- In the aging kidney, the interplay between immunosenescence and inflammaging promotes a pro-inflammatory microenvironment that impairs tissue repair and accelerates functional decline.

**What is the implication of the main finding?**

- Targeting inflammaging and senescence with senolytics, immunomodulation, and lifestyle interventions may represent promising strategies to extend healthspan and mitigate age-related diseases, particularly renal pathologies

**Abstract:**

Aging is characterized by a chronic, low-grade inflammatory state known as inflammaging, which closely interacts with immunosenescence—the gradual deterioration of immune function. Together, these processes contribute to tissue dysfunction and the development of age-related diseases. This review explores the cellular and molecular mechanisms underlying inflammaging, including mitochondrial dysfunction, telomere attrition, impaired autophagy, and gut microbiota dysbiosis. A particular emphasis is given to the senescence-associated secretory phenotype (SASP), which sustains a pro-inflammatory microenvironment and exacerbates tissue damage. We further discuss the impact of inflammaging on major age-related pathologies, with a focus on the kidney as a paradigmatic model of age-related decline, where inflammaging and cellular senescence contribute to chronic kidney disease (CKD) and impaired regeneration. Finally, we summarize emerging therapeutic strategies such as senolytics, senomorphics, immunomodulation, and lifestyle interventions, aimed at reducing the burden of senescent cells, mitigating inflammaging and extending healthspan.

## 1. Introduction

Inflammaging is a term introduced in the early 2000s by Prof. Franceschi to describe the chronic and systemic increase in inflammation levels that occurs with aging [1]. This was a revolutionary conceptualization of immune changes in response to lifelong stress. This allowed the continuous pro-inflammatory process to be viewed as an adaptation that could eventually lead to both beneficial and harmful consequences. In particular, this phenomenon is characterized by a low-grade, persistent inflammatory response that can contribute to the onset of various age-related chronic diseases, such as cardiovascular diseases, type 2 diabetes, osteoporosis, and Alzheimer’s disease. Numerous epidemiological studies demonstrate that a mild state of inflammation, indicated by high levels of inflammatory biomarkers such as C-reactive protein and interleukin-6 (IL-6), is associated with various aging phenotypes [2]. These include variations in body composition, energy production and its use, metabolic balance, immune system aging, and neuronal health. Understanding the mechanisms behind inflammaging, such as the role of mitochondrial dysfunction, cellular senescence, and microbiota changes, can allow the development of targeted interventions to counteract these inflammatory processes and significantly improve survival rate and reduce the burden of age-associated diseases. The importance of this field is underscored by research indicating that inflammatory markers like IL-6 and Tumor Necrosis Factor alpha (TNF-α) have been reported to predict mortality in elderly populations, suggesting that controlling inflammation could enhance longevity and quality of life [3].

**Figure 1:**
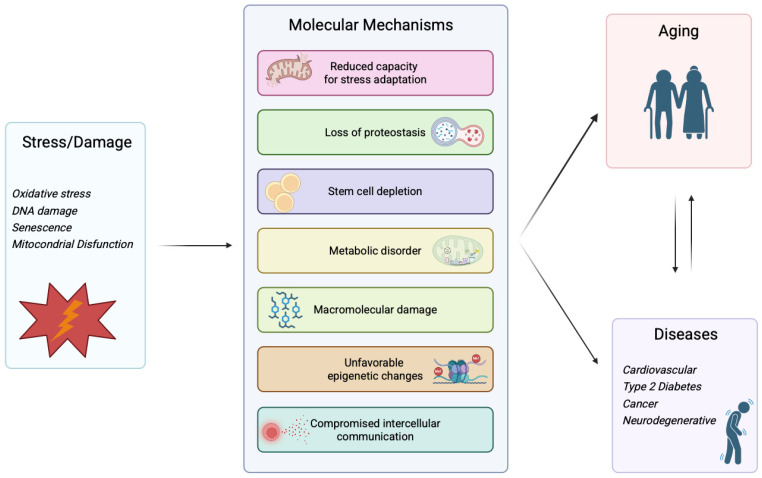
Schematic representation of the interconnection between stress/damage stimuli and age-related diseases. Various forms of stress and cellular damage—such as oxidative stress, DNA damage, senescence, and mitochondrial dysfunction—contribute to seven major molecular processes (the ‘pillars’ of aging), including reduced capacity for stress adaptation, loss of proteostasis, stem cell depletion, metabolic disorder, macromolecular damage, unfavorable epigenetic changes, and compromised intercellular communication. These interconnected mechanisms drive both the aging process and the onset of age-related diseases such as cardiovascular disorders, type 2 diabetes, cancer, and neurodegenerative diseases.

Geroscience is an evolving scientific field dedicated to exploring the biology of aging and its connection with age-related diseases. A central working hypothesis underlying this conceptual framework is that the mechanisms driving aging and diseases often largely overlap. In this context, seven common ‘pillars’ have been identified, representing the major biological processes of aging and the main associated research areas [4]: reduced capacity for stress adaptation, loss of proteostasis, stem cell depletion, metabolic disorder, macromolecular damage, unfavorable epigenetic changes, and compromised intercellular communication. It is worth noting that different conceptual frameworks have been proposed to categorize the biological processes of aging. Kennedy et al. introduced the concept of seven ‘pillars of aging,’ which highlight broad research areas such as proteostasis, stem cell function, and stress response [4], whereas López-Otín and colleagues, described a set of ‘hallmarks of aging’ that include genomic instability, telomere attrition, mitochondrial dysfunction, and altered intercellular communication [5,6]. Although the terminology and emphasis differ, both frameworks converge in depicting aging as the result of interconnected biological mechanisms rather than isolated defects (Figure 1).

There are essentially two opposing schools of thought regarding the nature of aging: the first one considers aging as a pathological state [7], while the other one views it as a physiological adaptation [8]. Despite these contrasting perspectives, both converge in recognizing aging as the most significant risk factor for most age-related diseases [9,10]. If aging is a disease, we could intervene to modulate the distinctive hallmarks with the aim of delaying chronic conditions, thereby increasing healthspan [11]. If aging is instead a natural physiological process and merely one among many risk factors, interventions to modify it may not lead to clinically significant reductions in disease incidence. Furthermore, even if it were feasible to decelerate the aging process, this might not necessarily affect the onset of chronic conditions. An alternative view suggests that aging and age-related chronic diseases share common mechanisms, influenced by genetic and environmental factors that differently modulate both the rate of aging and the emergence of these diseases [12].

Nevertheless, the underlying causes of inflammaging are still partly unknown, with various hypotheses proposed, including the accumulation of cellular debris. Among the proposed drivers, the ‘garb-aging’ theory, first introduced by Franceschi and colleagues in 2017 [13], suggests that macromolecules within cells progressively accumulate damage over time. In combination with the gradual decline of repair mechanisms and autophagy, this accumulation of cellular ‘waste’ can trigger inflammation through the innate immune system [1]. This theory is thus compatible with the hallmarks mentioned earlier [6]. Damaged cellular and organelle components, free radicals from oxidative stress, metabolites such as extracellular ATP, urate crystals, fatty acids, ceramides, peroxidized lipids, amyloid, cardiolipin, succinate, advanced glycation end-products, altered N-glycans [14] and High Mobility Group Box 1 (HMGB1), a nuclear protein that acts as a prototypical Damage-associated Molecular Pattern (DAMP), are recognized by a network of sensors as “danger” signals and initiate immune reactions that are necessary for physiological repair. However, as damage accumulates, the danger responses can become chronic and hence maladaptive [15]. While the accumulation of cellular waste plays a significant role in local and systemic inflammation, it is likely not the only factor driving inflammaging. In parallel, cellular senescence and immunosenescence fuel the inflammatory milieu. Cellular senescence refers to a state of permanent cell-cycle arrest triggered by stressors such as DNA damage, oxidative stress, or telomere shortening. Although these cells no longer proliferate, they remain metabolically active and secrete a complex mix of pro-inflammatory cytokines, chemokines, and proteases known as the Senescence-Associated Secretory Phenotype (SASP). Immunosenescence, instead, describes the age-related remodeling and functional decline of the immune system, encompassing both innate and adaptive arms. It involves reduced proliferative capacity of lymphocytes, impaired antigen presentation, and altered cytokine production, ultimately weakening host defense while contributing to chronic low-grade inflammation [16]. Although inflammation induced by the immune system is a critical defense mechanism against infections, there is surprisingly limited understanding of how immune system aging contributes to inflammaging and how the senescence of both innate and adaptive immune cells influences chronic inflammation associated with advanced age [17]. The interplay of these processes establishes a vicious cycle, particularly relevant for organs such as the kidney.

In this review, we critically examine the mechanisms of inflammaging, highlight current controversies, and discuss therapeutic opportunities to mitigate its impact on healthspan.

## 2. Cellular Mechanisms Contributing to Inflammaging

**Figure 2:**
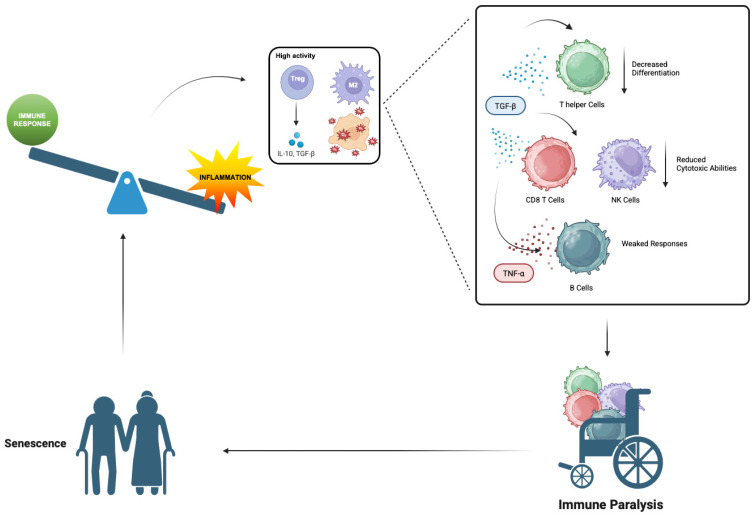
Immunosuppressive Shift in Aging: Treg Cells and Cytokine Balance. Aging promotes an immunosuppressive environment characterized by increased activity of regulatory T (Treg) cells and M2 macrophages results in the release of immunosuppressive factors, including IL-10 and TGF-β, which in turn impair T helper cell differentiation, reduce CD8^+^ T cell and NK cell cytotoxic abilities, and weaken B cell responses. This imbalance suppresses effective immune responses while sustaining chronic inflammation, contributing to immunosenescence and inflammaging. The diagram illustrates the disrupted equilibrium between immune activation and suppression in aged tissues, alongside increased production of reactive oxygen species such as superoxide (O_2_^−^), which further amplifies oxidative stress and inflammatory signaling. The overall consequence is a state of immune paralysis characterized by reduced immune competence and increased susceptibility to diseases.

During aging, the balance between inflammatory and immune responses becomes disrupted, leading to less efficient immune reactions and the creation of an immunosuppressive environment [18]. This shift is marked by an increase in certain cells like regulatory T (Treg) cells and M2 macrophages, which produce immunosuppressive factors such as transforming growth factor-beta (TGF-β), reactive oxygen species (ROS), and interleukin-10 (IL-10) (Figure 2).

IL-10 plays a key role in promoting the growth and activity of Treg cells and M2 macrophages, thereby enhancing immunosuppression [19]. These factors can also accelerate the aging of immune cells; for instance, TGF-β inhibits the differentiation of helper T (Th) cells, reduces the cytotoxic abilities of CD8 T cells and natural killer (NK) cells, and weakens B cell responses [20]. When this balance tips too far, ongoing inflammation can lead to “immune paralysis,” where the activation of T and B lymphocytes is compromised. This condition represents a feature of immunosenescence [21]. Although inflammation is clearly linked to immunosenescence, it is still debated whether it acts as a trigger or merely as a consequence of immune aging. [22]. Pro-inflammatory cytokines like tumor necrosis factor-alpha (TNF-α) can harm B cells and significantly lower antibody production, and prolonged inflammation can similarly lead to immune paralysis in macrophages and NK cells [23]. Here we will describe several sources of inflammaging which are not mutually exclusive but rather warrant further investigation.

**Figure 3:**
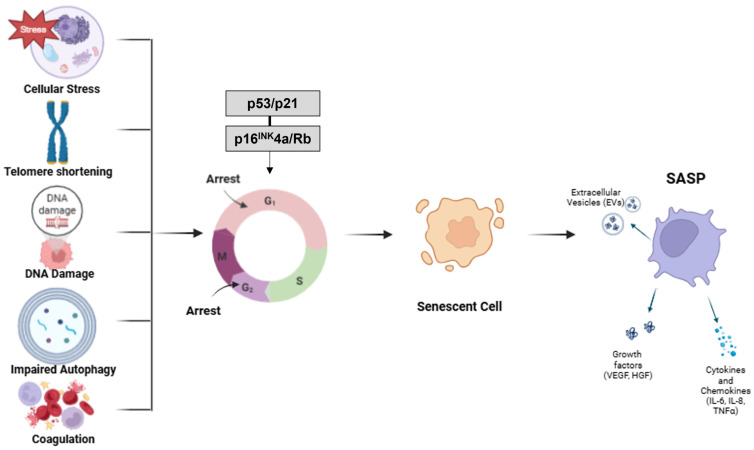
Cellular Mechanisms of Senescence and SASP Activation. Cellular senescence is triggered by a variety of stressors such as telomere shortening, DNA damage, mitochondrial dysfunction, impaired autophagy, and coagulation. These stimuli activate the p53/p21 and p16 ^INK^4a/Rb signaling pathways, leading to irreversible cell cycle arrest at distinct phases—G1 (first gap phase), S (DNA synthesis phase), G2 (second gap phase), and M (mitosis). Senescent cells, while no longer dividing, remain metabolically active and develop a senescence-associated secretory phenotype (SASP), characterized by the release of cytokines, chemokines, growth factors (such as VEGF and HGF), and extracellular vesicles (EVs) which contribute to a pro-inflammatory tissue environment. This chronic inflammation plays a central role in the progression of age-related pathologies and tissue dysfunction.

One of the most extensively studied contributors to inflammaging is cellular senescence, a state of permanent cell cycle arrest accompanied by distinct phenotypic and functional changes, which can be induced by various forms of cellular stress, in both proliferative and long-lived cells [24], including telomere shortening [25], DNA damage [26], and oncogene activation [27]. Senescent cells do not divide but remain metabolically active and secrete a complex mix of pro-inflammatory cytokines, growth factors, chemokines, and proteases, collectively termed the senescence-associated secretory phenotype (SASP) (Figure 3). The SASP significantly modifies the tissue microenvironment, promoting inflammation, altering the extracellular matrix, and influencing the behavior of neighboring cells [28]. For example, SASP may attract innate and adaptive immune cells near tumor cells [29] and a persistent secretion may cause chronic systemic inflammation and tissue damage and inhibit immune cell function in the elderly [30]. Accumulate evidence indicates also that SASP is not a fixed entity but highly heterogeneous and dynamic. Its composition depends on the cell type, the stimulus and the tissue microenvironment [31].

Moreover, several studies have shown that the SASP evolves over time: in the early stages it may include factors that promote tissue repair and immune clearance of senescent cells, whereas in later stages it shifts toward a more pro-inflammatory and fibrotic profile [32]. The diminishing capability of the immune system in clearing senescent cells is a significant factor contributing to inflammaging. In growing old, the immune system becomes less effective at recognizing and eradicating these cells, leading to their accumulation [33]. This persistent presence of senescent cells intensifies inflammatory responses due to ongoing SASP production [34]. Furthermore, senescent cells can induce senescence in neighboring cells via paracrine signaling, thereby amplifying the inflammatory milieu [35,36]. It was observed that elimination of senescent cells in prematurely aged mice prevents several age-related pathologies [37].

Moreover, senescent cells tend to accumulate at particularly high levels in adipose tissue, especially in the visceral fat [38]. Adipose tissue is also a significant source of inflammatory cytokines, and substantial changes in fat distribution, lipid composition, and function can have profound clinical implications, contributing to various age-related disorders [39]. Indeed, aging is associated with deep changes in lipid metabolism, characterized by an increase in triacylglicerol and lipoprotein in plasma, reduced ability to utilize lipids as energy substrates and increased ectopic lipid accumulation in liver, muscle, kidney and lungs [40]. Also, lipids serve as primary energy sources for cells and are efficiently stored within cytoplasmic organelles known as lipid droplets (LDs), which are insoluble or partially soluble in water [41]. The formation of LDs helps prevent the accumulation of toxic lipid species like fatty acids in the cytosol and reduces lipolysis and the subsequent production of ROS [42,43]. In cells with high exogenous lipid uptake, such as cancer cells, LDs function as lipid source that help prevent ferroptosis, a regulated form of cell death characterized by iron-dependent accumulation of lipid peroxides [44]. LDs are also crucial for cellular survival during periods of complete nutrient deprivation, as their breakdown provides substrates for mitochondrial β-oxidation [45]. In acute senescence, LD biosynthesis is coupled with increased mitochondrial β-oxidation, which may contribute to energy production, gene transcription or generation of lipid-derived senescence-related pro-inflammatory cytokines [46]. Additionally, LDs play a vital role in protecting cells from stress, and their biogenesis is upregulated as a conserved response to endoplasmic reticulum (ER) stress [47]. LDs can also help alleviate ER stress caused by hypoxia. During hypoxic conditions, protein synthesis is stopped, leading to the accumulation of misfolded proteins in the ER [48]. Hypoxic cells, including cancer cells, enhance their triacylglycerol uptake, biosynthesis, and LD formation through the activation of hypoxia-inducible transcription factor 1α (HIF-1α) [49]. Aging is associated with a general decline in tissue oxygenation and under such conditions, LDs may play a key role in mitigating hypoxia-induced proteasomal dysfunction by supporting ER function. Some evidence suggests that increasing lipid storage is necessary for the synthesis of pro-inflammatory mediators [50]. These studies provide support for the fundamental role of LDs in immune cell activation and maturation.

Another source of inflammaging could be harmful products from host microbes, such as those from the gut microbiota, which may also penetrate tissues and the bloodstream, causing inflammation [51]. The gut’s capacity to regulate these microbes declines with age, or the composition of the microbiota itself changes, leading to an inflammatory response [51,52]. In this context, probiotics and synbiotics are attracting increasing interest as potential modulators of inflammaging. By reshaping gut microbiota composition, strengthening intestinal barrier function, and reducing microbial translocation, these approaches may attenuate systemic inflammation and delay immunosenescence. Several preclinical and early clinical studies have reported that supplementation with probiotic strains (e.g., Lactobacillus, Bifidobacterium) can lower circulating pro-inflammatory cytokines and improve markers of metabolic health in older adults [53]. Recent reviews have further emphasized that microbiota-based interventions can influence not only metabolic and inflammatory pathways but also systemic immune homeostasis, suggesting a broader role for probiotics and symbiotics in the context of healthy aging [54]. Although evidence is still preliminary, targeting the gut–immune axis through microbiota-based interventions represents a promising complementary strategy in the management of age-related disorders. Chronic infections, such as cytomegalovirus, can also drive persistent immune activation and inflammation [55]; chronic inflammatory airway diseases, such as chronic rhinosinusitis with nasal polyposis, provide an example of how persistent immune activation and tissue remodeling can mirror the processes of inflammaging, highlighting the systemic relevance of local inflammatory conditions [56]. Furthermore, mitochondria play a major role in inflammaging and in the activation of NOD-like receptor family pyrin domain containing 3 (NLRP3) inflammasome, a cytosolic multiprotein complex that induces pro-caspase-1 self-cleavage and activation in response to cellular danger resulting in the maturation and secretion of the pro-inflammatory cytokines interleukin 1β (IL-1β) and interleukin 18 (IL-18) [57]. NLRP3 has been reported to be activated by a wide variety of unrelated Pathogen-Associated Molecular Pattern (PAMPs) and DAMPs, but there is no evidence that NLRP3 binds directly to these effectors. To date, multiple molecular and cellular events, including ion flux [58,59], mitochondrial dysfunction, the release of ROS and mitochondrial DNA (mtDNA) [60], lysosomal disruption and trans-Golgi disassembly [61], induced by NLRP3 stimuli have been proposed to be upstream signals for the assembly and activation of inflammasomes. Damage to mitochondrial DNA and the resultant production of ROS contribute to cellular damage and senescence [62]. As phylogenetically bacterial symbionts of early eukaryotic cells, damaged mitochondria release mitochondrial damage-associated molecular patterns (such as formyl peptides and mitochondrial DNA) into the circulation, which act as potent activators of innate immunity and NLRP3 inflammasome [63].

Telomere shortening is another significant contributor to cellular senescence and inflammaging. When telomeres reach a critically short length, cells enter a state of senescence [62]. Oxidative stress accelerates telomere attrition, linking it directly to mitochondrial dysfunction and the inflammatory environment seen in aging [64,65]. Telomerase is a ribonucleoprotein enzyme that counteracts the attrition and is essential for sustaining the proliferative capacity of dividing cells. In the immune system, telomerase activity is particularly important: it is transiently upregulated upon T and B cell activation to support clonal expansion by influencing key immunomodulatory factors such as NF-kB and β-catenin [66]; while its progressive decline with age contributes to immune exhaustion. Reduced telomerase activity in T lymphocytes is generally related with increased intracellular ROS and the accumulation of CD28-senescent T cells, which echibit shortened telomeres, low telomerase activity, high levels of pro-inflammatory cytokines and diminished production of antiviral cytokines [67]. Similar processes occur in B cells and NK cells, where telomere erosion impairs antibody production and cytotoxic activity, respectively [68,69]. Thus, telomere attrition and insufficient telomerase activity directly connect cellular senescence with immunosenescence, establishing a critical link to inflammaging. Moreover, the impairment of autophagy activity, the process by which cells remove damaged components, contributes to rich amount of dysfunctional cellular components and persistent inflammation. As a crucial cellular housekeeping process, autophagy helps maintain cellular homeostasis by facilitating the removal of dysfunctional organelles, such as mitochondria, and protein aggregates, thereby preventing the accumulation of pro-inflammatory DAMPs [70]. Impaired autophagy leads to the accumulation of damaged organelles and proteins, which can provoke inflammatory responses and contribute to the senescent phenotype [71] as decreased autophagic activity leads to increased oxidative stress and the activation of pro-inflammatory pathways, including NF-κB and inflammasomes [72]. Enhancing autophagic activity has been shown to attenuate age-related inflammation and improve cellular health, suggesting that therapeutic strategies aimed at modulating autophagy could help mitigate inflammaging and its associated pathologies [73].

Lastly, with advancing age, there is an increased activation of the coagulation system, leading to a hypercoagulable state which significantly elevates the risk of both arterial and venous thrombosis [74]. This hypercoagulability is associated with higher incidences of thrombotic events, such as deep vein thrombosis and pulmonary embolism, in the elderly population. The interplay between coagulation and inflammation further exacerbates this risk, highlighting the need for targeted therapeutic strategies to mitigate thrombotic complications in aging individuals [75].

In summary, inflammaging is driven by a complex interplay of mechanisms including the accumulation of senescent cells, immune system dysfunction, mitochondrial damage, telomere shortening, impaired autophagy, changes in the gut microbiome, and chronic infections. These factors collectively foster a chronic inflammatory state that promotes the development of age-related diseases, highlighting the importance of targeting these pathways for therapeutic interventions.

## 3. Decoding Inflammaging: Chronic Inflammation’s Role in the Onset and Progression of Age-Related Diseases

Pro-inflammatory molecules present in the bloodstream are significant predictors of age-associated morbidity and mortality [76]. Despite this, the extent to which systemic inflammatory factors act as primary drivers of age-related diseases in humans remains ambiguous. Emerging evidence suggests that local production of inflammatory cytokines within tissues may play a more critical role in driving the phenotypes and pathologies characteristic of aging. This concept is exemplified by the tumor microenvironment, where local inflammation significantly influences tumor progression and malignancy [77]. Similarly, the cytokine milieu within specific tissues is crucial in the pathogenesis of age-related retinal vascular diseases [78], highlighting the impact of localized inflammation on ocular health. Furthermore, the senescence-associated secretory phenotype (SASP) of damaged or senescent cells contributes to the deterioration of tissue architecture and function, underlining the role of localized inflammatory processes. Thus, elevated levels of inflammatory mediators in the circulation might often result from leakage from localized sources within tissues. Therefore, understanding the relative contributions of systemic versus local inflammation in the aging process is imperative. Nevertherless, the increasing number of senescent cells characterizes the old tissues during aging and age-related diseases (ARDs), including cardiovascular diseases (CVDs), type 2 diabetes (T2DM), musculoskeletal disorders, various types of cancer, and neurodegenerative diseases [79]. Senescence happens in both pathological and physiological processes, so we can consider it a double-edged sword because could provide positive effects in early life and negative ones later in life, in association with the accumulation of senescent cells in different tissues and organs. This could be explained by the ‘theory of antagonistic pleiotropy’ suggested by Williams in 1957 [80]: cellular damage and organismal aging are caused by pleiotrophic genes, or genes with multiple phenotypic effects. The accumulation of senescent cells promotes the immune system activation and the reduced senescent cells clearance is associated with the chronic immune induction; it turns out that this persistent response continuously fuels inflammaging. Thus, this notion is supported by studies showing that the selective elimination of senescent cells can extend the lifespan of genetically heterogeneous normal mice [81]. These findings have encouraged the search for senotherapeutics agents, which are compounds designed to promote the targeted removal of senescent cells [82]. Some of these treatments have been shown to delay the onset of age-related diseases and, as a result, extend the healthspan in mice [83], opening a new era in the field of geroscience with the creation of a new branch devoted to senotherapeutics.

One of the most well-established links between chronic inflammation and age-related disease is seen in cardiovascular diseases such as atherosclerosis and heart failure. Inflammaging contributes to the development of atherosclerotic plaques by promoting endothelial dysfunction and the accumulation of lipid-laden macrophages in the arterial walls [84]. Inflammatory markers like C-reactive protein (CRP) and interleukin-6 (IL-6) have been associated with the progression of atherosclerotic plaques and the destabilization of these plaques [85]. This inflammatory environment facilitates the progression of plaques, leading to coronary artery disease, heart attacks, and strokes. IL-6 and tumor necrosis factor-alpha (TNF-α) are often elevated in these conditions and serve as mediators of the inflammatory response. Indeed, inflammaging contributes to endothelial dysfunction, increased arterial stiffness, and the development of hypertension, which are significant risk factors for CVDs. Recent reviews have comprehensively discussed the mechanisms of cardiovascular inflammaging, highlighting the interplay between senescent cells, mitochondrial dysfunction, and immune aging in the vascular system [86]. Together, these insights underscore inflammaging as a central determinant of cardiovascular vulnerability during aging.

Furthermore, in the central nervous system, chronic inflammation is a significant contributor to neurodegenerative diseases such as Alzheimer’s and Parkinson’s. In Alzheimer’s disease, the accumulation of amyloid-beta (Aβ) plaques and tau tangles is associated with a heightened inflammatory response, involving microglial activation and the release of neurotoxic cytokines, which exacerbate neuronal death [87], contributing to cognitive decline. Increased Aβ deposition can be captured by local antigen-presenting cells (APCs) in the brain, leading to the activation and expansion of Aβ-reactive T cells, ultimately causing brain inflammation [88]. Alzheimer’s disease patients have fewer naive T cells, a higher number of memory T cells, and significantly shortened telomeres in T cells [89,90]. Similarly, in Parkinson’s disease (PD), both the innate and adaptive immune systems lose competence with aging and are notably altered: inflammation is linked to the degeneration of dopaminergic neurons in the substantia nigra [91], with elevated levels of inflammatory mediators like IL-1β and TNF-α being observed in affected brain regions [92]. In the context of neurodegeneration, the concept of neuroinflammaging captures how aging-induced immune alterations and neuroinflammation are coupled to accelerate neuronal damage. Indeed, with aging microglia acquire a senescent or ‘primed’ phenotype, losing efficient phagocytic capacity, adopting dystrophic morphology, and secreting increased levels of pro-inflammatory cytokines—changes that contribute to the chronic inflammatory milieu in the brain [93]. These observations stress that, in the brain, inflammaging is not just systemic but intimately linked with local cellular aging processes.

Likewise, metabolic disorders, including type 2 diabetes and obesity, are also tightly linked to chronic inflammation. Inflammaging is associated with insulin resistance, a key feature of type 2 diabetes, where pro-inflammatory cytokines such as IL-1β and IL-6 interfere with insulin signaling pathways [94]. This impairment in glucose metabolism results in hyperglycemia and its associated complications, such as neuropathy, nephropathy, and retinopathy. Adipose tissue in obesity becomes infiltrated with immune cells, particularly macrophages, which secrete pro-inflammatory mediators, exacerbating metabolic dysfunction and further driving systemic inflammation [95]. Moreover, endocrine alterations may further modulate immune and inflammatory responses during aging, as suggested by the association between thyroid function and transplant outcomes [96], reinforcing the concept of an integrated immuno-endocrine axis in age-related vulnerability. Lastly, the metabolic disorders in immune cells lead to the dysregulation of nicotinamide adenine dinucleotide (NAD+) metabolism leading to activation inflammatory pathways and acceleration of immunosenescence [97]. Franceschi and colleagues have extensively reviewed this perspective, proposing that low-grade chronic inflammation acts as a shared pathogenic substrate linking obesity, diabetes, and age-related diseases through common metabolic and immune pathways [3].

Beyond metabolic dysfunction, chronic inflammation is increasingly recognized as a significant factor in the development and progression of various cancers. Inflammation can create a microenvironment that promotes tumor growth, survival, and metastasis, by inducing DNA damage, promoting angiogenesis, and altering immune responses [98]. As highlighted earlier, inflammatory cells can release cytokines and chemokines such as TNF-α and IL-6 which facilitate tumor proliferation and the evasion of apoptosis, allowing cancer cells to survive and proliferate unchecked. Studies have shown that chronic inflammation is associated with many types of cancer, including hepatocellular carcinoma, colorectal cancer, and gastric cancer. Among these, prostate disease in aging men illustrates how chronic inflammation of the tissue microenvironment may favor carcinogenesis, supporting the role of inflammaging as a permissive background for tumor initiation [99]. Inflammatory mediators, such as prostaglandins, can suppress the immune response against tumors, leading to an increased risk of cancer development and progression [100,101]. Recent research has focused on understanding the molecular mechanisms linking chronic inflammation to cancer. For instance, the identification of TREM1+ regulatory myeloid cells in hepatocellular carcinoma highlights the role of specific immune cell subsets in creating a pro-tumorigenic environment through immune suppression [102]. Additionally, the expression of hydroxyprostaglandin dehydrogenase in regulatory T cells has been shown to enhance immunosuppressive effects in chronic inflammation, further linking these processes to cancer progression [103]. By modulating the inflammatory microenvironment, it may be possible to enhance anti-tumor immune responses and improve cancer therapy outcomes.

The intricate interplay between chronic inflammation and age-related diseases underscores the importance of targeting inflammatory pathways as a therapeutic strategy. Efforts to modulate inflammaging through lifestyle interventions, pharmacological agents, and novel therapeutics hold promise for improving healthspan and reducing the burden of age-related diseases. Future research should focus on identifying precise biomarkers of inflammaging and understanding the molecular mechanisms underlying this process to facilitate the development of effective interventions [104].

Aging is a major risk factor for a wide spectrum of chronic diseases; however, current evidence indicates that cardiovascular diseases (CVDs), type 2 diabetes mellitus (T2DM), and neurodegenerative disorders such as Alzheimer’s and Parkinson’s disease exert the most profound impact on aging trajectories. While inflammaging contributes to the pathogenesis of cardiovascular, neurodegenerative, metabolic disorders, and cancer, the kidney represents a paradigmatic model of age-related vulnerability. As a highly vascularized organ with continuous exposure to circulating cytokines, danger signals (DAMPs), and microbial products (PAMPs), the kidney is particularly sensitive to chronic inflammatory stress. Moreover, renal aging manifests early through nephron loss, fibrosis, and impaired regenerative capacity. For these reasons, the kidney provides a unique setting to study the convergence of immunosenescence, inflammaging, and tissue dysfunction, which will be the focus of the next section.

## 4. The Impact of Cellular Senescence on Kidney Aging and Pathophysiology

Senescence is triggered by various stimuli, including telomere attrition, DNA damage, oxidative stress, and mitochondrial dysfunction. Senescent cells are characterized by a distinct secretory phenotype, known as the senescence-associated secretory phenotype (SASP), which includes pro-inflammatory cytokines, growth factors, and proteases [105]. These secreted factors contribute to a pro-inflammatory environment, influencing tissue remodeling and function. In the kidneys, the accumulation of senescent cells interferes with normal cellular function, leading to fibrosis and defective renal regeneration [106]. The concept of “inflammaging” describes the chronic, low-grade inflammation that support aging, heightened by the presence of senescent cells. Inflammaging is particularly damaging in the kidneys, as these organs are highly vascularized and exposed to systemic inflammatory mediators. The persistent inflammation associated with inflammaging enhances the progression of chronic kidney disease (CKD) and other renal pathologies in the elderly [107]. Sepe et al. emphasize the significance of pattern recognition receptors (PRRs), including Toll-like receptors (TLRs), NOD-like receptors (NLRs), and others, in mediating inflammatory responses in the kidneys. These receptors recognize PAMPs and DAMPs, triggering immune responses that can lead to CKD and other renal dysfunctions [108]. Very important is the overexpression of TLRs in various kidney conditions, such as autoimmune diseases, acute kidney injury (AKI), and transplant rejection, which accelerates the progression of renal inflammation and fibrosis [109,110,111]. TLR signaling, particularly through MyD88/TRIF (Myeloid differentiation primary response 88-Toll/IL-1 Receptor Domain-Containing Adapter-Inducing Interferon-β) pathways, results in the activation of pro-inflammatory cytokines like IL-6 and TNF-α, exacerbating kidney damage [112]. Also, the involvement of other PRRs, such as C-type lectin receptors (CTLRs) and RIG-I-like receptors (RLRs), promote inflammation and contribute to renal aging [113].

In the aging kidney there is a marked accumulation of senescent cells in various renal compartments, including the glomeruli and renal tubules. This accumulation correlates with a decrease in nephron numbers, the development of glomerulosclerosis, and tubulointerstitial fibrosis, which all contribute to the lower glomerular filtration rate (GFR) observed in elderly individuals [114]. Senescent cells in the kidney release SASP factors, which promote inflammation and fibrosis, further exacerbating renal damage and functional decline [115]. The presence of markers such as senescence-associated beta-galactosidase (SA-β-Gal), p16 (known also as INK4a), and p21 (Kip1), have consistently been found to be increased in aged kidneys and also in response to kidney injury, as observed in various animal models and human renal conditions [116]. Studies have shown that these senescence markers are elevated in rodent models of hypertension induced by DOCA-salt (Deoxycorticosterone Acetate-salt), diabetic nephropathy (DN) caused by streptozotocin (STZ), and cisplatin-induced nephrotoxicity [117,118,119]; particularly in DN, senescence markers like SA-β-Gal and p16 are associated with clinical parameters such as BMI, LDL cholesterol, and blood glucose levels. These findings extend to human renal diseases, including hypertension, DN, chronic kidney disease (CKD), delayed graft function (DGF) post-transplantation, and several glomerular diseases such as membranous nephropathy, minimal change disease, IgA nephropathy (IgAN), and focal segmental glomerulosclerosis (FSGS) [120,121,122]. Notably, the accumulation of senescent cells correlates with renal histopathological changes and functional decline, including proteinuria and blood pressure abnormalities [123]. Senescent cells are predominantly localized in the renal cortex, particularly in proximal tubular cells, though they are also found in glomerular, interstitial, and vascular cells depending on the stressor involved [124,125]. Cellular senescence is implicated in the pathogenesis of various age-related kidney diseases, including CKD and acute kidney injury (AKI). In CKD, senescence-induced inflammation and fibrosis accelerate disease progression and increase the risk of cardiovascular complications [126]. Fibrosis in renal damage and renal aging arises from a complex interplay of pathological processes and signaling pathways, including pro-inflammatory and fibrotic signaling, the depletion of renoprotective factors like Klotho and bone morphogenetic proteins, vascular rarefaction, and oxidative stress [127] and cellular senescence is a central process connecting existing mechanisms of fibrosis. Macrophages, particularly the M1 and M2 subtypes, play a crucial role in driving inflammation and fibrosis in the kidneys. Indeed, the chronic activation of these immune cells leads to persistent renal inflammation, which contributes to tissue damage and fibrosis [128]. In AKI, senescent cells impair renal repair mechanisms, leading to incomplete recovery and increased susceptibility to subsequent injuries [129]. Immunosenescence, as age-related decline in immune function, further complicates the aging process by reducing the body’s ability to clear senescent cells and increasing susceptibility to infections and chronic diseases [130]. Premature immunosenescence has been observed in patients with CKD, leading to an immune system that is functionally older than the patient’s chronological age. This condition contributes to the high morbidity and mortality rates associated with CKD, particularly in the elderly [131]. Similarly, the recurrence of glomerulonephritis after kidney transplantation underscores the contribution of chronic immune dysregulation [132].

Recent research has focused on developing therapeutic strategies to target senescent cells and mitigate their harmful effects on the kidney. Senolytic drugs, which selectively eliminate senescent cells, have shown promise results in preclinical models, improving renal function and reducing fibrosis [133,134]. Additionally, caloric restriction and pharmacological modulation of SASP components are being explored to delay renal aging and preserve kidney function [135,136]. The interaction between cellular senescence and inflammaging creates a vicious cycle that drives the progression of renal aging and pathology. Furthermore, targeting the innate immune system, specifically through the inhibition of PRRs or cytokines like IL-6, could be a promising therapeutic strategy for managing renal inflammaging and preventing the progression of age-related kidney diseases. For instance, experimental models have shown that blocking TLR2 can reduce inflammation and fibrosis in the kidneys [137,138], highlighting the potential for translational applications in human treatments.

In this review, we highlight the role of the innate immune system in kidney inflammaging and the potential of targeting it to mitigate age-related renal decline. Chronic activation of pattern recognition receptors (PRRs) and innate immune cells contributes directly to renal dysfunction and fibrosis, and experimental inhibition of pathways such as TLR2 or HMGB1 has been shown to reduce inflammation and limit fibrotic changes. Understanding the molecular mechanisms driving senescent cell accumulation and associated inflammaging remains essential for developing targeted strategies to preserve kidney function during aging.

## 5. Perspectives on Current Research and Ongoing Therapies

Current research on inflammaging is exploring new molecular and cellular pathways that could serve as therapeutic targets. Advanced genomic and proteomic techniques are helping identify biomarkers of inflammaging. Among the ongoing therapies, the modulation of the immune response through therapeutic vaccines and the use of senolytics, which selectively eradicate senescent cells, represent promising approaches. In addition, senomorphics, compounds that suppress the pro-inflammatory signaling of senescent cells without killing them, can reduce the harmful effects of senescent cells, mitigating chronic inflammation. These therapies have shown potential in preclinical studies for reducing inflammation, improving tissue repair, and slowing the progression of age-related diseases [139,140]. Additionally, lifestyle interventions such as caloric restriction and increased physical activity are being explored for their ability to reduce inflammaging and promote healthy aging. Senolytics are a class of drugs that target the senescent cells’ resistance to apoptosis, which is maintained by the activation of specific pro-survival pathways [141]. By clearing these cells, senolytics aim to reduce the SASP molecules, thereby mitigating chronic inflammation and its detrimental effects on tissue function, including in the kidneys. Preclinical studies have demonstrated that senolytics can reduce renal inflammation and fibrosis, potentially slowing the progression of chronic kidney disease (CKD) specifically inducing apoptosis of senescent tubular epithelial cells [142,143]. Pharmacological approaches have also emerged as promising strategies. For instance, inhibiting kidney-type glutaminase effectively eradicated senescent cells in aged mice, improving kidney function and reducing inflammation [144,145]. Another approach concerns the use of a modified peptide Forkhead Box O4 (FOXO4-DRI) to interfere wiht the interaction between p53 and FOXO4, inducing apoptosis in senescent cells [82]. This method decreased senescent cell burden in aged kidneys, enhancing renal function and reducing inflammatory markers. Further, small molecule senolytics repurposed from oncology, have shown potential in experimental models of aging. The flavonoid fisetin, for example, demonstrated a capacity to reduce senescent cells, diminish SASP gene expression, and preserve renal histology [146], with its mitochondrial protective effects likely contributing to these benefits. Another senolytic, ABT-263, a potent B cell lymphoma (Bcl)2/w/xL inhibitor, targets BCL-2 family members and depletes senescent cells when administered orally to irradiated or normally aged mice and has been shown to improve kidney repair, and attenuate fibrosis in aged mice [147]. Moreover, the combination of quercetin and dasatinib (DQ) has progressed to clinical trials, where it has shown promise in reducing senescent cell markers and circulating SASP factors in diabetic patients [148], and improving renal markers in non-human primates. This combination also enhanced the expression of the anti-aging protein α-Klotho in both mice and human patients, suggesting its potential in ameliorating age-related kidney dysfunction [149]. However, dasatinib and quercetin target different components of the anti-apoptotic pathways that senescent cells rely on. Dasatinib, a tyrosine kinase inhibitor, and quercetin, a flavonoid with anti-inflammatory properties, have been shown to decrease the burden of senescent cells in various tissues. The effects of dasatinib and quercetin treatment together were observed also in amyloidogenic Alzheimer’s disease mouse model [150]. The mice exhibited increased oxygen consumption and energy expenditure, leading to a reduction in body mass. Additionally, white adipose tissue mass decreased, along with senescence markers, SASP, blood glucose, and plasma levels of insulin and triglycerides.

The interest in senolytics has led to several clinical trials aimed at evaluating their safety and efficacy in humans: UNITY Biotechnology’s ubx0101, a small molecule designed to clear senescent cells in knee osteoarthritis and had advanced to phase 2 [151]; Dasatinib and quercetin in Idiopathic Pulmonary Fibrosis, a trial which has completed its early phases, showed that short-term treatment with these drugs reduced the number of senescent cells in the lungs and decreased markers of inflammation [152]. The outcomes could guide future studies on senolytics, particularly in how these drugs might be used to delay the incidence of multiple age-related conditions.

While senomorphics may offer a safer profile by avoiding cell loss, senolytics have the advantage of clearing the source of SASP entirely. Furthermore, senomorphics do not remove the senescent cell burden, which remains a limitation compared to senolytics, and in some cases they may have immunosuppressive effect. Several classes of senomorphics have been proposed each acting on specific cellular pathways involved in inflammaging. Rapamycin and its analogs (rapalogs) inhibit the mammalian Target Of Rapamycin (mTOR), a central regulator of cell growth and metabolism, thereby promoting autophagy and reducing SASP expression well in the immune, vasculare and integumentary systems [153]. Sirtuin-1 (SIRT1) activators such as resveratrol, enhance mitochondrial function and genomic stability while modulating inflammatory gene transcription [154]. p38-mitogen activated protein kinase (p38-MAPK) inhibitors block stress-induces signaling cascades that amplify SASP production [155], whereas Janus kinase/signal transducer and activator of transcription (JAK/STAT) inhibitors interfere with cytokine-mediated communication, weakening systemic inflammation [156]. Ataxia-Telangiectasia Mutated (ATM) inhibitors target the DNA damage response pathway, which is chronically activated in senescent cells and contributes to SASP maintenance [157]. Finally, metabolic modulators improve cellular energy balance and reduce oxidative stress, indirectly mitigating senescence-related dysfunction [157]. While preclinical data suggest benefits on inflammation, tissue remodeling, and lifespan, clinical translation is still limited, and most ongoing human trials have focused on senolytics rather than senomorphics, highlighting a gap in translational studies.

To better illustrate the therapeutic landscape of inflammaging interventions, Table 1 summarizes key compounds currently under investigation, including both senolytics and senomorphics.

## 6. Conclusions

Continued research and clinical trials are essential to validate senolytic and senomorphic therapies. The results of ongoing studies will be crucial for refining the therapeutic strategies and may clear the way for the integration of senolytics into standard care for age-related diseases, including those affecting the kidneys. Indeed, while the elimination of senescent cells has shown beneficial effects in animal models, the long-term efficacy and safety of senolytics are uncertain.

Despite these advancements, challenges remain in translating preclinical findings into effective clinical therapies. Limitations of the current work include the reliance on preclinical models and the impossibility of fully capturing the multifactorial complexity of inflammaging in humans. Future research should prioritize a deeper understanding of molecular mechanisms underlying inflammaging and the identification of reliable biomarkers, potentially combining omics approaches with extracellular vesicle analysis, to stratify patients and monitor therapeutic responses. Finally, personalized approaches that consider the variability in the aging process among individuals may also enhance the effectiveness of these actions. Clinical trials need to evaluate not only pharmacological strategies, such as senotherapeutic agents, but also lifestyle and microbiota-based interventions, including probiotics and symbiotics. A critical roadmap for the field should therefore integrate these complementary approaches, aiming to delay multiple age-related diseases simultaneously and to extend healthspan rather than lifespan alone.

## Figures and Tables

**Table 1:** Comparison of current anti-inflammaging agents based on their cellular targets and translational potential, with emphasis on renal and systemic effects observed in preclinical and clinical studies.

Compound/Class	Type	Mechanism of Action	Evidence Level	Notes
**Dasatinib + Quercetin (DQ)**	Senolytic	Targets anti-apoptotic pathways; clears senescent cells	Preclinical + Clinical	Reduces SASP, improves renal markers, increases α-Klotho expression
**Fisetin**	Senolytic	Flavonoid; reduces senescent cells and SASP gene expression	Preclinical	Preserves renal histology; mitochondrial protective effects
**ABT-263 (Navitoclax)**	Senolytic	BCL-2 family inhibitor; induces apoptosis in senescent cells	Preclinical	Improves kidney repair and reduces fibrosis
**FOXO4-DRI peptide**	Senolytic	Disrupts FOXO4-p53 interaction; induces apoptosis in senescent cells	Preclinical	Enhances renal function and reduces inflammatory markers
**Kidney-type glutaminase inhibitor**	Senolytic	Eradicates senescent cells in aged kidneys	Preclinical	Improves kidney function and reduces inflammation
**UBX0101 (UNITY Biotechnology)**	Senolytic	Small molecule targeting senescent cells in osteoarthritis	Clinical (Phase 2)	Reduces senescent cells and inflammation in joints
**Rapamycin and Rapalogs**	Senomorphic	mTOR inhibition; suppresses SASP without killing cells	Preclinical	May have immunosuppressive effects; limited clinical translation
**Resveratrol**	Senomorphic	SIRT1 activator; modulates SASP and inflammation	Preclinical	Natural compound; low bioavailability
**p38-MAPK inhibitors**	Senomorphic	Inhibits inflammatory signaling pathways	Preclinical	Potential for tissue remodeling
**JAK/STAT inhibitors**	Senomorphic	Blocks cytokine signaling; reduces SASP	Preclinical	May affect immune function
**ATM inhibitors**	Senomorphic	Modulates DNA damage response and SASP	Preclinical	Still experimental
**Metabolic modulators**	Senomorphic	Various pathways; aim to reduce SASP and improve tissue function	Preclinical	Broad category; clinical data lacking

## Data Availability

No new data were created or analyzed in this study. Data sharing is not applicable to this article.

## Abbreviations

The following abbreviations are used in this manuscript:

ATM	Ataxia-Telangiectasia Mutated
AKI	Acute Kidney Injury
BMI	Body Mass Index
CKD	Chronic Kidney Disease
CRP	C-Reactive Protein
DAMP	Damage-associated molecular pattern
DGF	Delayed Graft Function
DN	Diabetic Nephropathy
DNA	Deoxyribonucleic Acid
DOCA	Deoxycorticosterone Acetate
DRI	Disrupting p53–FOXO4 interaction (FOXO4-DRI peptide)
ER	Endoplasmic Reticulum
FOXO4	Forkhead Box O4
FSGS	Focal Segmental Glomerulosclerosis
GFR	Glomerular Filtration Rate
HIF-1α	Hypoxia-Inducible Factor 1-alpha
HMGB1	High Mobility Group Box 1
IL-6	Interleukin-6
JAK	Janus Kinase
LD	Lipid Droplets
LDL	Low-Density Lipoprotein
mTOR	Mammalian Target of Rapamycin
MyD88	Myeloid differentiation primary response 88
NAD^+^	Nicotinamide Adenine Dinucleotide
NF-κB	Nuclear Factor kappa-light-chain-enhancer of activated B cells
NK	Natural Killer cells
NLR	NOD-like receptor
NLRP3	NOD-like receptor family pyrin domain containing 3
NOD	Nucleotide-binding oligomerization domain
PAMP	Pathogen-Associated Molecular Pattern
PD	Parkinson’s Disease
RIG-I	Retinoic acid-inducible gene I
ROS	Reactive Oxygen Species
SA-β-Gal	Senescence-Associated β-Galactosidase
SASP	Senescence-Associated Secretory Phenotype
SIRT1	Sirtuin-1
STAT	Signal Transducer and Activator of Transcription
STZ	Streptozotocin
TGF-β	Transforming Growth Factor Beta
TLR	Toll-Like Receptor
TLR2	Toll-Like Receptor 2
TNF-α	Tumor Necrosis Factor Alpha
TREM1	Triggering Receptor Expressed on Myeloid Cells 1
TRIF	Toll/IL-1 Receptor Domain-Containing Adapter-Inducing Interferon-β
